# The species of the genus *Hypodynerus* de Saussure (Hymenoptera, Vespidae, Eumeninae) occurring in Brazil

**DOI:** 10.3897/zookeys.296.4794

**Published:** 2013-04-29

**Authors:** Bolívar R. Garcete-Barrett, Marcel Gustavo Hermes

**Affiliations:** 1 Laboratório de Biologia Comparada de Hymenoptera, Departamento de Zoologia, Universidade Federal do; 2 Paraná, Cx. Postal 19020, 81531-980 Curitiba, PR, Brazil; 3 Setor de Zoologia, Departamento de Biologia, Universidade Federal de Lavras, Cx. Postal 3037, 37200-000 Lavras, MG, Brazil

**Keywords:** Vespidae, Brasil, neotropics, identification key, taxonomy

## Abstract

An identification table and descriptions are given to recognize the two species of *Hypodynerus* (Hymenoptera: Vespidae: Eumeninae) recorded from Brazil: *Hypodynerus arechavaletae* (Brèthes) and *Hypodynerus duckei* (Bertoni) **comb. n.** The lectotype is designated and the male is described for *Hypodynerus duckei*, its presence being recorded from Brazil for the first time.

## Introduction

*Hypodynerus* is a fairly large South American genus of potter wasps with nearly 50 presently recognized species (Barrera Medina 2011). Its species are distributed from Colombia to Southern Chile (Willink 1970), mostly along the Andes and westward, and with a decreasing number of species in the biogeographical provinces of Patagonia, Monte, Pampas, Chaco and the Parana subregion. Just two species are recorded from the latter area, which are the only two species found up to now in Brazil.

The species of *Hypodynerus* are recognizable by having the tergum 1 without a transverse carina but with a well-marked preapical longitudinal sulcus, the male sternum 7 with a marked medial longitudinal channel, the anterior pronotal face devoid of coarse macropunctation or medial foveae, but with two shallow and widely separated transverse submedial impressions and the propodeum without upper lamellae, without a separate dorsal horizontal surface and with a well-developed medial carina not preceded by a fovea; propodeal valvulae rather small, gently rounded and completely fused to the submarginal carina. They also share the following characters: Maxillary palpi 6-segmented; labial palpi 4-segmented; female without cephalic foveae; pronotum without oblique humeral carina and well developed pretegular carina; epicnemial carina undeveloped; regularly spindle-shaped tegula with the hind apex pointed and emarginated, embracing the parategula; prestigma short; second submarginal cell basally acute and anteriorly sessile; axilla simple and posteriorly falling perpendicularly into the axillary fossa; scutellar crest not lamellate over the axillary fossa; axillary fossa wide open; metanotum simple and metsomal terga and sterna with simple apical margin (Carpenter and Marques 2001, Carpenter and Garcete-Barrett 2003, Sarmiento and Carpenter 2006).

Willink (1970) separated seven species groups (*humeralis*, *ruficollis*, *excipiendus*, *arechavaletae*, *labiatus*, *caupolicanus* and *chiliotus*) and revised the first of the groups. In two subsequent papers (Willink 1978a and 1978b) he revised the following two groups, but up to now the remaining four groups, representing the bulk of the genus, are still unrevised, although some scattered new species, belonging to those groups, have been described since then (Willink 1981, Willink and Lobo 1992, Pérez 1999, Barrera Medina 2011).

## Material and methods

This paper is mostly based on material deposited in the entomological collection of the Departamento de Zoologia, Universidade Federal do Paraná (DZUP) and some additional material including the types of *Nortonia duckei* Bertoni from the invertebrates collection of the Museo Nacional de Historia Natural del Paraguay (IBNPY), as well as some additional specimens kindly loaned to us by Jucélia Iantas from the Universidade Estadual do Centro-Oeste, UNICENTRO.

A few abbreviations have been used in the descriptions: F, T and S accompanied by a number refer to antennal flagellomeres and metasomal terga and sterna respectively.

### The Brazilian species of *Hypodynerus* de Saussure

The Brazilian *Hypodynerus* belong in the *arechavaletae* group, which was characterized by Willink (1970) as having a rather campanulate, relatively narrow and dorsally evenly convex, not-angulate T1, and S2 basally convex to flattened, without any kind of tubercle or angulation. They are medium sized wasps with rather simple propodeum, male antennae and male mid-legs, and with a general body shape resembling some species of *Pachymenes* or *Pachyminixi*. This species group is the most important one representing the genus to the east of the Andes and its species are, in general, less hairy than in most of the other species groups. The genitalia of both species found in Brazil is peculiar in bearing a pecten of coarse setae on the basivolsella, above a field of sparser setae, and by having a mostly bare digitus, with some thin setae just along the ventral margin. The species are separable by the characters given in Table 1.

**Table 1.** Separation of the species.

***Hypodynerus arechavaletae* (Brèthes)**	***Hypodynerus duckei* (Bertoni)**
1) Pronotum visibly bent longitudinally along the humeral region and humeral angle marked as seen in posterior oblique view.	1) Pronotum gently rounded longitudinally along the humeral region, without a well-marked humeral angle as seen in posterior oblique view.
2) T1 shorter: 0.88 times as broad as long in females and with a slightly shorter petiole (ratio of pre-spiracular : post-spiracular section = 7:10), clearly campanulated in males.	2) T1 longer: 0.73 times as broad as long in females and with a slightly longer petiole (ratio of pre-spiracular : post-spiracular section = 8:10), more gradually widening behind in males.
3) Mesonotal admedial line a narrow shallow depression.	3) Mesonotal admedial line raised into a thin carina.
4) Propodeum bent along between the lateral and posterior surfaces.	4) Propodeum rather rounded-off between the lateral and posterior surfaces.
5) Propodeal concavity with at most some reflective pilosity very close to the submarginal carina as seen from above.	5) Propodeal concavity covered with reflective pilosity over most of its surface.
6) Brownish infuscation along costal region of the wing paler basad of pterostigma, brightly amberish there.	6) Brownish infuscation along costal region dark all along, specially basad of pterostigma.
7) Metasoma with yellow apical bands, a narrow one on T1 and moderately broad ones on the following terga and sterna.	7) Metasoma with at most (males) a narrow yellow apical band on T1 and gradually thinner and weaker, medially fainter, bands on T2-T6 and S2-S6, being them thinner and testaceous to absent in females.
8) Mesosoma all dark in both sexes.	8) Mesosoma with lemon-yellow (males) to testaceous (females) markings including: hind pronotal margin, upper mesepisternal spot (absent in females examined), scutellar crests, lateral scutellar spots (absent in females examined) and transverse metanotal band.
9) Females with at most a large dorsal reddish area on all tibiae, and darkly reddish apex of femora.	9) Females with legs all reddish apically from the apex of femora.

## Taxonomy

### Hypodynerus arechavaletae

(Brèthes)

http://species-id.net/wiki/Hypodynerus_arechavaletae

Figs 1 and 3

Odynerus arechavaletae Brèthes, 1903: 285, 317 (key), female, male (in subgenus *Odynerus* division *Hypodynerus*) - “Argentina; Uruguay” (Museo de Ciencias Naturales Bernardino Rivadavia, Buenos Aires?).
Nortonia pilifrons Kohl, 1907: 239, 248, pl. 8 figs. 28, 29, 31, 34, female, male - “Brasilien (Rio Grande do Sul ...)” (Naturhistorisches Museum, Wien). - Zavattari, 1912: 166 (syn. of *Nortonia arechavaletae* (Brèthes)).
Nortonia arechavaletae ; Zavattari, 1912: 166 (syn.: *Nortonia pilifrons* Kohl; Argentina; Uruguay; Brazil). - Brèthes, 1920 (1919): 396 (Argentina).
Hypodynerus arechavaletae ; Willink, 1970: 235 (key). - Willink and Chiappa, 1993: 120 (doubtful for Chile). - Barrera Medina, 2011: 161 (absent from Chilean collections).

#### Description.

**Female**: Body length 12.8–14.4 mm. Wing length 11.9–12.5 mm. Background color *brownish-black* with *orange-yellow* markings as follows: an orbital spot just above the clypeus, a very brief orbital line on the upper temple, a very thin marginal line on T1, a broader marginal line (diffusely continued at sides on T2) on T2-T5 and S2-S5, a broad submarginal line on T6 and a large central spot on S6. *Dark reddish* are: a central spot on the tegula and a broad outer line on all of the tibiae (anterior on fore tibia) which does not reach the apex. Wings *light-brown* stained, darker at the marginal cell and *brightly amberish* tinged along the costal region basad of the pterostigma. Pterostigma and costal venation *amberish*, rest of the venation *dark chestnut*.

Clypeus elongate, about 1.23 times longer than broad, with a moderately raised and flattened disc and the apex a little broader than the interantennal distance and subtruncate, very shallowly emarginate between rounded lateral teeth. Temples rounded. Pronotal carina right angled at humeri as seen posterolaterally. Mesonotal admedial line a narrow shallow depression. Parapsidal lines well marked. Notauli well marked along the posterior third. Scutellum slightly tuberculated mesoanteriorly. Propodeum bent along between the lateral and posterior surfaces, sides rather flat as seen from behind. T1 broadly campanulate and with a slightly short petiole (0.88 times as broad as long and ratio of pre-spiracular : post-spiracular section = 7:10).

Clypeus subshining, with moderate micropunctation separated by 2–4 diameters, all covered with sparse and thin macropunctures and just some coarser and denser ones at the apex. Head and mesosoma covered with moderately dense macropunctation which gets absolutely thin and sparse behind the ocelli and is much denser and partly coalescent at the very suprahumeral area. Tegula dull, densely micropunctate and with some scattered thin macropunctures along its inner margin. Metepisternal surface with just very few scattered thin macropunctures and with some short longitudinal striae anteriorly. Propodeum coarsely striatopunctate on its upper half at sides and behind, becoming thinly striate and sparsely macropunctate bellow. Disc of T1 covered with scattered macropunctures. T2-6 and S2-6 dull and with just hardly visible, very scattered microscopic macropunctures.

Whole body covered with pale golden-brown apressed pile. Head, mesosoma, T1, S1 and S2 also with long, pale yellowish-brown, erect hairs. Propodeal concavity with at most some reflective pilosity very close to the submarginal carina as seen from above.

**Male**: Body length 10.7 mm. Wing length 9.4 mm. Coloration and structure as described for the female but: without reddish color on the legs and with a broad yellow line along the clypeus. F11 yellowish dorsally and F8-F10 reddish ventrally. T7 mostly yellow and S7 reddish basally at middle. Clypeus shorter, more convex and more definitely notched between acute teeth. Flagellomeres not especially elongate and hardly with dorsal knobs. F8 with a deep semicircular ventro-apical notch. Sensillae of F8 and F10 small and nearly circular. Sensilla of F9 about twice as large and irregularly circular, with a more or less flat side basally. T1 not too different in shape to that of the female.

**Figures 1–2.**
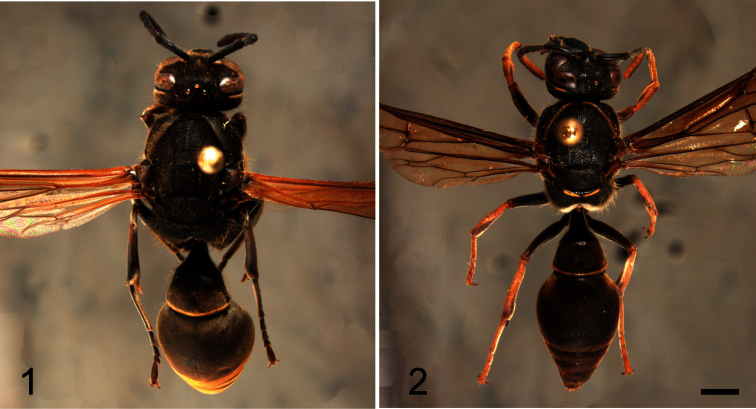
Female habitus in dorsal view. **1** *Hypodynerus arechavaletae* (Brèthes) **2** *Hypodynerus duckei* (Bertoni). Scale bar = 1 mm.

**Figures 3–4.**
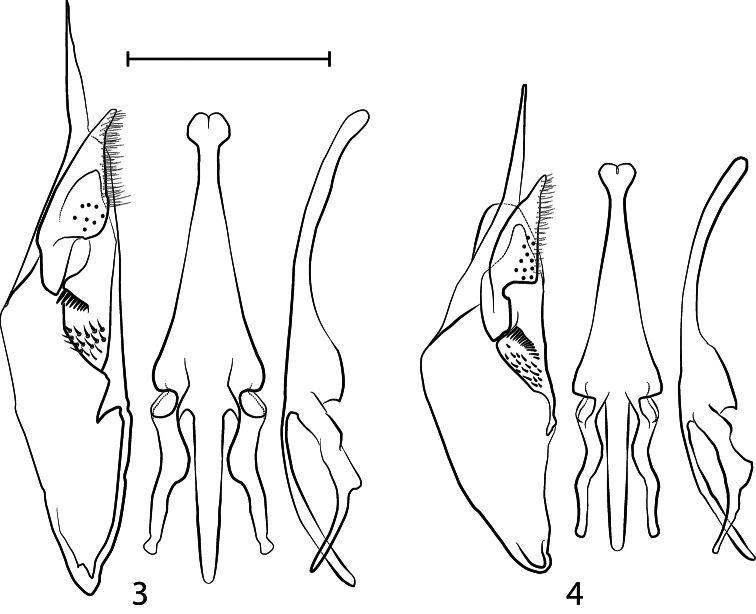
Male genitalia, showing the right paramere and volsella in lateral inner view to the left, the aedeagus in ventral view at the middle, and the aedeagus in lateral view to the right **3** *Hypodynerus arechavaletae* (Brèthes)  **4** *Hypodynerus duckei* (Bertoni). Scale bar for all the figures = 1 mm.

#### Material examined.

8 females and 1 male as follows: BRAZIL: Paraná: 40 km E Palmas, 26°33'S, 51°36'W, 1280 masl, 18.xi.2009 (*G. Melo, K. Ramos & V. Kanamura*) [1 male: DZUP]; Piraquara, Mananciais da Serra, 18.x.2011 (*P.C. Grossi*) [4 females: DZUP]; same locality, 18.xii.2006 (*M.G. Hermes*) [1 female: DZUP]; same locality, 23.xi.2005 (*E. Carneiro*) [2 females: DZUP]; São Paulo: 10 km SW São José Barreiro, Serra da Bocaina, 22.721°S, 44.625°W, 1560 masl, 24.x.2011 (*M.G. Hermes*) [1 female: DZUP].

#### Biology.

Unknown.

### Hypodynerus duckei

(Bertoni) comb. n.

http://species-id.net/wiki/Hypodynerus_duckei

Figs 2 and 4

Nortonia duckei Bertoni, 1918: 194, 207, female - [Paraguay] “Mondaíh” (Museo Nacional de Historia Natural del Paraguay, San Lorenzo). Lectotype here designated [EXAMINED].

#### Description. 

**Female**: Body length 11.0–13.1 mm. Wing length 10.2–12.2 mm. Background *color brownish black*, with *testaceous* to *orange-* or *lemon-yellow* markings as follows: an orbital spot just above the clypeus, a very brief orbital line on the upper temple, very thin hind pronotal margin, pronotal lobe (very darkly), very dark and mostly absent small subalar spot, scutellar and metanotal crests, a transverse and briefly medially interrupted metanotal band (absent in most of the specimens), a very thin marginal line on T1 (even weaker medially) and rather obscure marginal bands on T2-T5. *Dark reddish* are: pronotal lobe (occasionally and very dark), a very small subalar spot (in just one specimen), sides of T1 behind and below the spiracle, a reduced latero-basal marginal region of T2, almost imperceptible lateral indications of apical band on T2-T5 and all of the legs dorsally from the very apex of the femur to the last tarsomere. Wings light brown stained, darker along all of the costal region, including the marginal cell. Pterostigma *amber brown* and venation *dark chestnut*.

Clypeus elongate, about 1.25 times longer than broad, with a moderately raised and flattened disc and with the apex hardly broader than the interantennal distance and subtruncate, very shallowly emarginate between rounded lateral teeth. Temples rounded. Pronotal carina rounded off at humeri, not angled at all, as seen posterolaterally. Mesonotal admedial line raised into a thin carina. Parapsidal lines well marked. Notauli well marked along the posterior fourth. Scutellum very slightly elevated mesoanteriorly, but not tuberculate. Propodeum rather rounded-off between the lateral and posterior surfaces, sides gently convex as seen from behind. T1 elongate campanulate, with a slightly longer petiole than in *Hypodynerus arechavaletae* (0.73 times as broad as long in females and ratio of pre-spiracular : post-spiracular section = 8:10).

Clypeus subopaque, with moderate micropunctation separated by 1-3 diameters, all covered with sparse and thin macropunctures and just some coarser and denser ones at the apex. Head and mesosoma covered with moderately dense macropunctation which gets thin and sparse behind the ocelli and are much denser and partly coalescent on the upper pronotal face and an anterolateral area of the mesoscutum. Tegula subshining, moderately micropunctate and with very few scattered thin macropunctures along its inner margin. Metepisternal surface with a number of moderately scattered macropunctures and longitudinally striate on its anterior half. Propodeum coarsely striatopunctate on its upper half behind, sparsely striatopunctate above at sides and becoming thinly striate and sparsely macropunctate bellow. Disc of T1 covered with moderately dense macropunctures. T2-6 and S2-6 dull and with just hardly visible, very scattered microscopic macropunctures.

Whole body covered with brown appressed pile. Head, mesosoma, T1, S1 and S2 also with long, pale yellowish, erect hairs. Propodeal concavity covered with reflective pilosity over most of its surface.

**Male**: Body length 8.4–8.7 mm. Wing length 7.9–8.0 mm. Coloration and structure as described for the female but: without reddish color on the legs, with all of the flagellum orange ventrally, with all of the yellow markings sharper and brightly *lemon-yellow* and including: a large apical spot on the clypeus, inner orbital markings linear and reaching the opening of the ocular notch, posterior margin of pronotum, subalar spot, lateral subanterior spot on scutellum, scutellar crest, complete metanotal band, a thin and clear marginal band on T1-T4 and S2, and more obscure (and gradually more obscure posteriorly) ones on T5-T6 and S3-S6. F2-F8 strongly nodular anteriorly. Clypeus shorter, more convex and more definitely notched between acute teeth. Flagellomeres elongate and F1-F8 with a progressively stronger dorsal knob. F8 shallowly emarginated ventro-apically. Sensilla of F8 and F9 large, elyptical and about equal in size. Sensilla of F10 very small and circular. Disc of T1 more elongate than in female.

#### Material examined.

32 females and 2 males as follows: TYPE MATERIAL: PARAGUAY: [Alto Paraná]: “Alto Mondaih” (labeled by Bertoni as “Typus” and with the sequential number 3053, and by Garcete with the sequential number AWB-F129) [1 female LECTOTYPE HERE DESIGNATED: IBNPY]; same locality (labeled by Bertoni with the sequential number 3053 and by Garcete with the sequential numbers AWB-F163, AWB-F164, AWB-F165 and AWB-F146) [4 females PARALECTOTYPES: IBNPY]. ADDITIONAL MATERIAL: BRAZIL: Paraná, Guaratuba, 25°52'14"S, 48°58'04"W, 30.vii.2005 (L.R.R. Faria) [1 female: DZUP]; Piraquara, Mananciais da Serra, 30.xi.2005 (M.G. Hermes) [3 females: DZUP]; same locality, 22.xi.2005 (M.G. Hermes) [2 females: DZUP]; same locality, 9.xii.2005 (P.C. Grossi) [3 females: DZUP]; same locality, 4.xii.2006 (M.G. Hermes) [8 females: DZUP]; same locality, 30.x.2006 (P. Grossi) [1 female: DZUP]; same locality, 10.x.2007 (M. Hermes) [1 female: DZUP]; same locality, xi.2005 (P.C. Grossi) [1 female: DZUP]; same locality, 18.xii.2006 (M.G. Hermes) [1 female: DZUP]; same locality, 3.xi.2002 (A.J.C. Aguiar, G. Melo & J. Rozen); same locality, 25°29'S, 48°58'W, 8.xi.2008 (P.C. Grossi) [1 female: DZUP]; same locality, 25°29'S, 48°59'W, 1050 masl, 30.xii.2005, collecting mud (G. Melo) [1 female: DZUP]; Santa Catarina: Porto União, 17.ii.2012, emerged from trap nest (J. Iantas) [1 female: DZUP; 1 female: UNICENTRO]; 3.ii.2012, same locality, emerged from trap nest (J. Iantas) [1 female, 2 male: DZUP; 1 male: UNICENTRO].

#### Biology.

Unknown.

## Supplementary Material

XML Treatment for Hypodynerus arechavaletae

XML Treatment for Hypodynerus duckei
